# Increased risk of all-cause, Alzheimer’s, and vascular dementia in adults with migraine in Korea: a population-based cohort study

**DOI:** 10.1186/s10194-022-01484-y

**Published:** 2022-08-24

**Authors:** Kyungduk Hurh, Sung Hoon Jeong, Seung Hoon Kim, Suk-Yong Jang, Eun-Cheol Park, Sung-In Jang

**Affiliations:** 1 Department of Preventive Medicine, Yonsei University College of Medicine, 50-1 Yonsei-ro, Seodaemun-gu, Seoul, 03722 Republic of Korea; 2 Institute of Health Services Research, Yonsei University, Seoul, Republic of Korea; 3 Department of Public Health, Graduate School, Yonsei University, Seoul, Republic of Korea; 4 Department of Healthcare Management, Graduate School of Public Health, Yonsei University, Seoul, Republic of Korea

**Keywords:** Migraine, Chronic headaches, Dementia, Alzheimer’s dementia, Vascular dementia, Risk-set matching

## Abstract

**Background:**

Studies investigating the association between migraine and dementia have reported inconsistent findings. This study aimed to evaluate whether patients with migraine have an increased risk of dementia compared to individuals without migraine.

**Methods:**

We obtained data from the 2002–2019 Korean National Health Insurance Health Screening Cohort. Non-migraine controls were selected using a 1:1 risk-set matching with a time-dependent propensity score. The main outcome was the development of all-cause dementia, and the secondary outcome was the development of each cause of dementia (Alzheimer’s, vascular, mixed or other specified, and unspecified dementia). The incidence rate of dementia was calculated using Poisson regression, and the association between migraine and dementia was evaluated using Cox proportional hazards regression.

**Results:**

Among 88,390 participants, 66.1% were female, and the mean baseline age was 55.3 ± 9.4 years. During the study period, dementia cases were identified in 4,800 of the 44,195 patients with migraine and 3,757 of the 44,915 matched controls. The incidence rate of dementia was 139.6 (95% confidence interval [CI], 135.7–143.5) and 107.7 (95% CI, 104.3–111.1) cases per 10,000 person-years in patients with migraine and matched controls, respectively. Patients with migraine had a 1.30 (hazard ratio [HR], 1.30; 95% CI, 1.25–1.35), 1.29 (HR, 1.29; 95% CI, 1.23–1.35), 1.35 (HR, 1.35; 95% CI, 1.19–1.54), 1.36 (HR, 1.36; 95% CI, 1.00–1.83), and 1.30 (HR, 1.30; 95% CI, 1.17–1.45) times higher risk of developing all-cause dementia, Alzheimer’s dementia, vascular dementia, mixed or other specified dementias, and unspecified dementia than their matched controls, respectively.

**Conclusion:**

Our results suggest that migraine is associated with an increased risk of subsequent dementia. Further research is warranted to confirm these findings and to reveal the underlying mechanisms.

**Supplementary Information:**

The online version contains supplementary material available at 10.1186/s10194-022-01484-y.

## Background

Migraine is a common primary headache disorder characterized by episodic disabling headaches and is often accompanied by focal neurological symptoms called aura [1]. Migraine affects approximately 15% of the general population worldwide and ranks second among the top causes of disability [2, 3]. While migraine is most prevalent in young and middle-aged adults, dementia, another leading cause of disability, primarily affects the geriatric population [4].

Previous studies investigating the association between migraine and the risk of dementia have shown inconsistent results. Several cohort studies have reported an increased risk of all-cause dementia, Alzheimer’s dementia (AD), or vascular dementia (VaD) in patients with migraine compared to non-migraine individuals, while others did not find such relationships [5–8]. Moreover, in two studies, an association between migraine and the risk of dementia was observed only in women [9, 10]. In addition, studies on the relationship between migraine and cognitive function have shown inconsistent findings [11–13]. The heterogeneity of the results among studies may be attributed to differences in their designs. Specifically, the identification of patients with migraine (incident cases or retrospectively evaluated at baseline), duration of follow-up (5–24 years), the age distribution of the study population, and outcomes (all-cause dementia, AD, or VaD) varied among the studies.

Considering the long preclinical stage of AD and the gap between the prevalent age of migraine and dementia, a sufficient follow-up period should be evaluated to ensure the longitudinal relationship between the two disorders [14]. Additionally, since each cause of dementia (e.g., AD and VaD) has a different pathophysiology, separate analyses investigating the association between migraine and each type of dementia should be performed. A sufficient sample size is required to secure the statistical power to perform separate analyses for each type of dementia. Another research interest is migraine aura, a known risk factor for various conditions, including ischemic stroke and myocardial infarction [15–17]. Islamoska et al. reported that migraine with aura (MA) was associated with an increased risk of dementia in a Danish cohort study [7]. However, there is still insufficient evidence for the association between MA, migraine without aura (MO), and dementia among the Asian population.

Therefore, this study aimed to investigate the risk of developing all-cause dementia, AD, VaD, and other specified and unspecified dementias after migraine diagnosis over a 16-year follow-up period using Korean population-based data. To assess the temporal context of the association between migraine and dementia, additional analyses were performed according to the age at migraine diagnosis and follow-up duration. Finally, based on the findings of previous studies, we evaluated whether the presence of aura or sex affects the association between migraine and dementia.

## Methods

### Study subjects and data sources

We used data from the 2002–2019 Korea National Health Insurance Service Health Screening Cohort (NHIS-HEALS). All Korean citizens aged ≥ 40 years are eligible for the biennial general health screening program. The NHIS-HEALS comprised 514,866 general health screening participants aged between 40 and 79 years in 2002, equating to a 10% simple random sample of the target population [18]. The data were provided by the National Health Insurance Service (NHIS), which covers 97% of the Korean population. Since the NHIS also manages the healthcare claims of the remaining 3% of the Korean population, the medical aid program beneficiaries, the NHIS database contains the medical records of the entire Korean population. The NHIS-HEALS includes anonymized participant information (sex, age, health insurance premium decile determined by household income, residential areas, medical records, and health screening database). All participants were followed up until their loss of eligibility due to death or emigration [18].

### Migraine cohort

The migraine cohort was constructed as follows: First, individuals with at least two diagnoses of migraine (International Statistical Classification of Diseases and Related Health Problems, 10th revision [ICD-10] G43) during the study period were assigned to the migraine cohort. Next, patients with medical records of migraine from January 1, 2002, to December 31, 2003 (a two-year washout period), were excluded. The migraine cohort was further subdivided into the MA group (ICD-10 code G43.0), MO group (ICD-10 code G43.1), and unspecified group (none of the above).

### Identification of dementia cases

The primary outcome was the incidence of all-cause dementia. The secondary outcomes were each cause of dementia, including AD (ICD-10 codes G30 or F00), VaD (ICD-10 code F01), other specified dementias (ICD-10 codes F02, G31.00, G31.82), and unspecified dementia (ICD-10 code F03). Individuals were classified as dementia patients if they had at least two ambulatory visits or one hospital admission for dementia and did not have any medical records of dementia before December 31, 2003.

### Risk-set matching with propensity score

This study tried to mimic the prospective study design and overcome the inherent limitations of the retrospectively constructed NHIS-HEALS through the risk-set matching method using time-dependent propensity score [19, 20]. The control group was selected from individuals at risk of migraine, considering other potential confounders such as age, sex, socioeconomic status, comorbidities and lifestyle factors.

First, we calculated the hazard component for being patients with migraine represented as a time-dependent propensity score, using a Cox proportional-hazard model with January 1, 2004 as baseline (after the 2002–2003 washout period) and migraine diagnosis as an event [21]. The baseline characteristics of the participants used for propensity score matching were collected over two years before the baseline (2002–2003). Age (continuous variable), sex, household income level (medical aid beneficiary or decile for NHIS enrollees), residential area (urban or rural), registered disability, past medical history (stroke, diabetes mellitus, hypertension, and depression; individuals with more than two outpatient visits or one admission based on ICD-10 codes), smoking status (never smoker, ex-smoker, current smoker, or unspecified), BMI (< 25 or ≥ 25), and alcohol consumption (none, ≤ 7, 7–14, or > 14 units per week) were included as covariates.

Second, each patient with migraine was matched to individuals of the same age, sex, baseline household income level, and at risk of migraine at the index date (the date of criteria for migraine cohort were fulfilled). The index dates of the control individuals were set the same as the index dates of their matched patients with migraine. We repeated this risk-set matching sequentially for all migraine patients [22]. To mimic the design of a prospective study, risk-set matching was performed independently of the future diagnosis of migraine. In other words, because the control individuals were those who had not yet developed migraine (at risk of migraine) at the time of matching, they had the possibility of developing migraine during follow-up. Therefore, most individuals who had been assigned to the migraine cohort before matching were included as patients with migraine in the main analysis, but a minority were included as controls for other patients who developed migraine before them.

Subsequently, a 1:1 matching on time-dependent propensity score was performed for each risk set using a nearest-neighbor matching algorithm with a maximum difference of hazard components between patients with migraine and control individuals of < 0.1 [19, 23]. The matched patient-control set was removed from the subsequent risk set to generate a non-overlapping sample. The 1:1 propensity score matching was repeated until no more patients with migraine remained in the risk set.

### Statistical analyses

The balance of baseline characteristics between the migraine and control groups was assessed with a standardized difference; if the absolute value of the standardized difference was less than 0.1, the distribution of covariates was considered balanced [24]. We used the Kaplan–Meier method and stratified log-rank test to evaluate the cumulative incidence of dementia in patients with migraine and matched controls. We calculated the incidence rate (IR, the number of dementia cases per 10,000 person-years) of dementia and the 95% confidence interval (CI) using a generalized estimating equation with a Poisson distribution. The effect size was estimated as the hazard ratio (HR) using the Cox proportional hazards model. Subgroup analyses according to age at migraine diagnosis, follow-up duration, sex, and presence of aura were also performed. In some cases, the first date of migraine diagnosis during study period might be indicative of an active period of migraine (instead of new migraine onset), particularly among patients who were initially diagnosed with migraine at an older age. Therefore, we performed additional sensitivity analyses that investigated the association between migraine and dementia (all-cause, AD, VaD, mixed or other specified, and unspecified dementia) among individuals with first migraine diagnosis at age < 60 years. Moreover, same analyses were performed with five years of washout period (instead of two years) to exclude more active period of previously diagnosed migraine. Statistical significance was defined as a two-tailed *p*-value of < 0.05. All analyses were performed using the SAS Enterprise Guide software (version 7.1; SAS Institute, Cary, NC, USA) and R (version 4.1.3; Vienna, Austria; Rproject.org/).

## Results

**Fig. 1:**
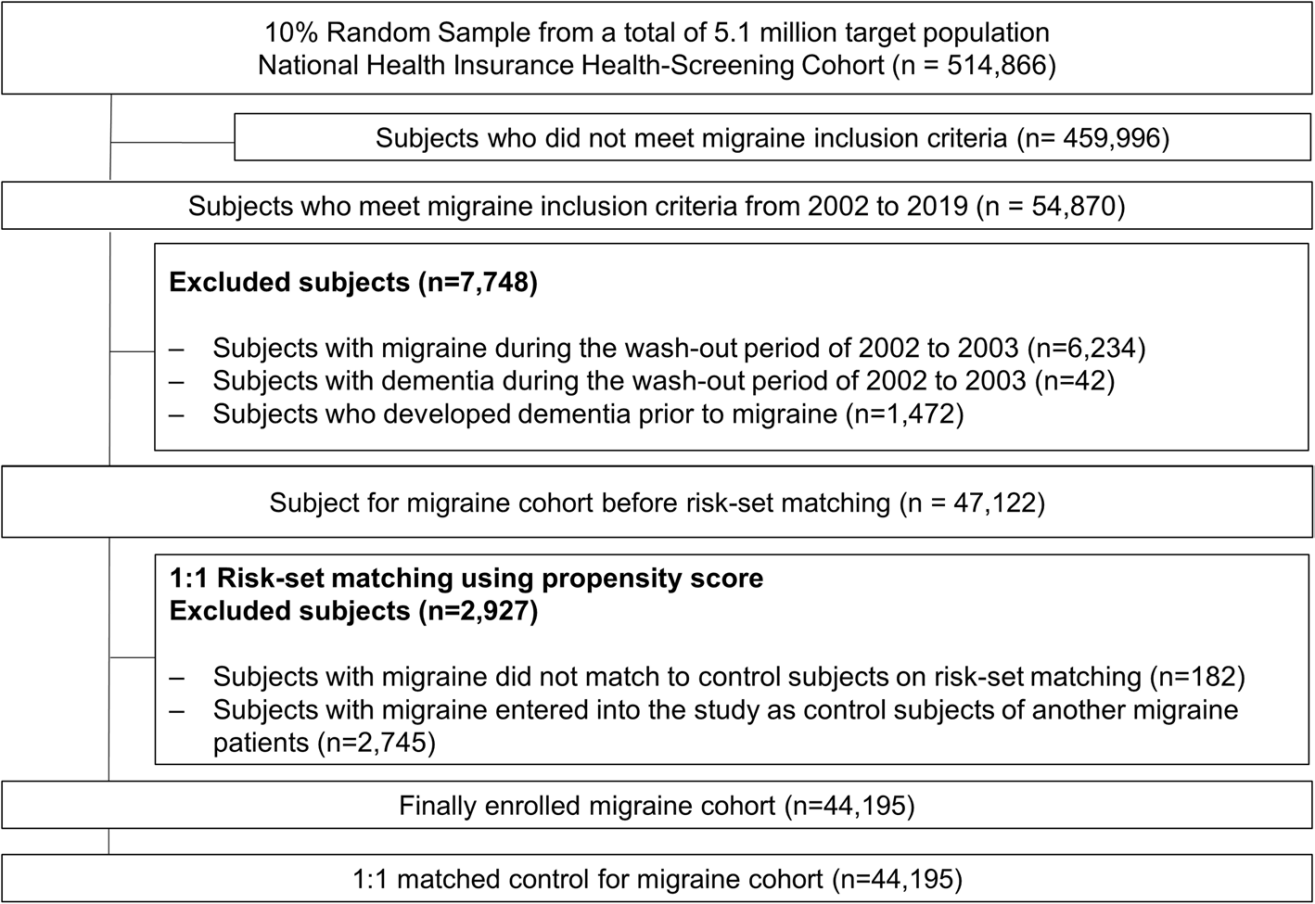
Flowchart of participant selection

Between January 1, 2002, and December 31, 2019, 54,870 individuals met the inclusion criteria for the migraine cohort. Of these, 10,675 individuals were excluded because they had medical records for migraine (*n* = 6,234) or dementia during the washout period (*n* = 42), developed dementia before migraine diagnosis (*n* = 1,472), were included as controls for other patients with migraine (*n* = 2,745), or showed insufficient matching results (*n* = 182). The final study sample comprised 44,195 patients with migraine and 44,195 matched controls (Fig. 1). The mean follow-up time was 7.84 years (maximum, 16 years), and 692,867 person-years were generated. There were 8,557 patients with new-onset dementia during the 16-year follow-up period. In the baseline year of 2004, 66.1% of the study subjects were female, and the mean age was 55.3 (± 9.4) years. The standardized differences for all covariates were < 0.1 (Table 1).

**Table 1:** Baseline characteristics of the study population

Characteristics	Patients, No. (%)		Standardized Difference
	**Migraine cohort (*n* = 44,195)**	**Matched controls (*n* = 44,195)**	
**Sex**			
Male	14,967 (33.9)	14,967 (33.9)	0
Female	29,228 (66.1)	29,228 (66.1)	
**Age, mean, (SD), y**	55.3 (9.4)	55.3 (9.4)	0
**Household income level**			
Medical aid program	369 (0.8)	369 (0.8)	0
First	3,942 (8.9)	3,942 (8.9)	
Second	3,361 (7.6)	3,361 (7.6)	
Third	3,461 (7.8)	3,461 (7.8)	
Fourth	3,454 (7.8)	3,454 (7.8)	
Fifth	3,509 (7.9)	3,509 (7.9)	
Sixth	3,732 (8.4)	3,732 (8.4)	
Seventh	4,352 (9.8)	4,352 (9.8)	
Eighth	4,960 (11.2)	4,960 (11.2)	
Ninth	6,255 (14.2)	6,255 (14.2)	
Tenth	6,800 (15.4)	6,800 (15.4)	
**Residential area**			
Urban	17,510 (39.6)	17,629 (39.9)	0.006
Rural	26,685 (60.4)	26,566 (60.1)	
**Registered disability**			
No	44,037 (99.6)	43,932 (99.4)	0.035
Yes	158 (0.4)	263 (0.6)	
**History of stroke**			
No	43,040 (97.4)	43,024 (97.4)	0.002
Yes	1,155 (2.6)	1,171 (2.6)	
**History of ischemic heart disease**			
No	43,683 (98.8)	43,563 (98.6)	0.024
Yes	512 (1.2)	632 (1.4)	
**History of diabetes mellitus**			
No	42,852 (97)	42,566 (96.3)	0.036
Yes	1,343 (3)	1,629 (3.7)	
**History of hypertension**			
No	41,923 (94.9)	41,805 (94.6)	0.012
Yes	2,272 (5.1)	2,390 (5.4)	
**History of antidepressant use (> 90 days)**			
No	36,189 (81.9)	35,772 (80.9)	0.024
Yes	8,006 (18.1)	8,423 (19.1)	
**Smoking status**			
Never-smoker	30,989 (70.1)	30,675 (69.4)	0.019
Ex-smoker	5,327 (12.1)	5,400 (12.2)	
Current-smoker	6,895 (15.6)	7,036 (15.9)	
Unspecified	984 (2.2)	1,084 (2.5)	
**BMI, kg/m**^**2**^			
< 25	29,397 (66.5)	29,069 (65.8)	0.021
≥ 25	14,768 (33.4)	15,076 (34.1)	
Unspecified	30 (0.1)	50 (0.1)	
**Drinking, units/weeks**			
None	29,937 (67.7)	29,706 (67.2)	0.013
≤ 7	7,147 (16.2)	7,276 (16.5)	
8–14	2,856 (6.5)	2,847 (6.4)	
> 15	3,317 (7.5)	3,393 (7.7)	
Unspecified	938 (2.1)	973 (2.2)	

**Fig. 2:**
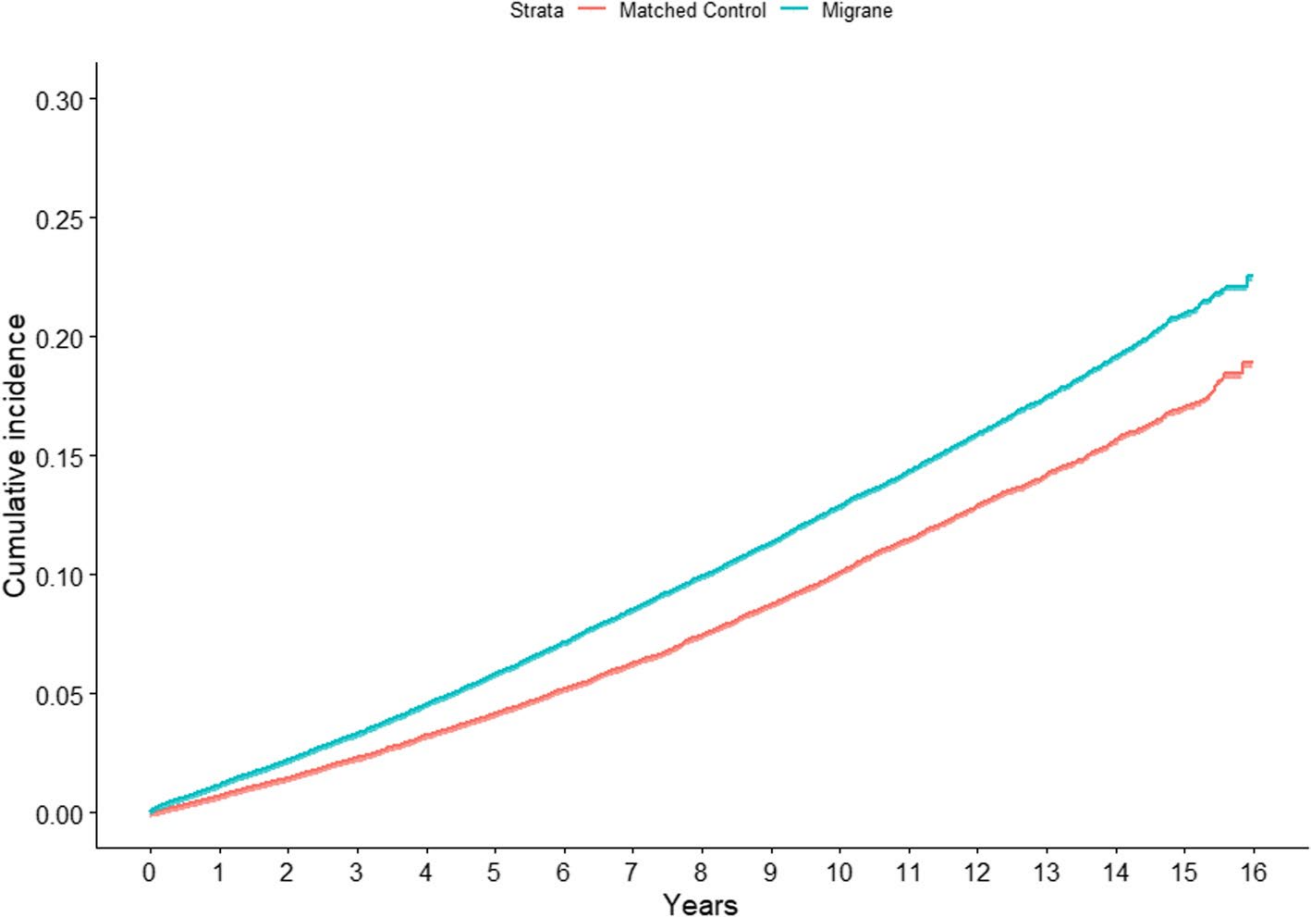
Cumulative incidence of all-cause dementia in migraine patients and their risk set-matched controls during follow-up. *p*-values for stratified log-rank tests < 0.001

The cumulative incidence of dementia during the entire study period showed a statistically significant difference between the migraine and control cohorts (*p* < 0.001, stratified log-rank test; Fig. 2).

Among the 44,195 patients with migraine, 4,800 developed dementia over 343,871 person-years (IR, 139.6 per 10,000 person-years; 95% CI, 135.7–143.5), while 3,757 developed dementia over 348,995 person-years (IR, 107.7 per 10,000 person-years; 95% CI, 104.3–111.1) among the 44,195 control subjects. Patients with migraine were 1.30 times more likely to develop dementia than their matched controls (HR, 1.30; 95% CI, 1.25–1.35). Patients with migraine showed 1.42 times (HR, 1.42; 95% CI, 1.34–1.51), 1.26 times (HR, 1.26; 95% CI, 1.14–1.40), and 1.16 times (HR, 1.16; 95% CI, 1.05–1.28) higher risk of developing dementia than their matched controls at 0–5, 6–10, and > 10 years after migraine diagnosis, respectively. However, the magnitudes of association between migraine and risk of dementia were similar between sexes, between groups by age at migraine diagnosis, and among groups by the presence of aura (Table 2).

**Table 2:** Comparable analysis for the association between migraine and the risk of all-cause dementia

Variables	Subjects, No	Dementia cases, No	Person- Years, No	Incidence rate (95% CI) per 10,000 person years	Hazard ratio (95% CI)
**Full cohort**					
Matched Controls	44,195	3,757	348,995	107.7 (104.3–111.1)	1.00
Migraine cohort	44,195	4,800	343,871	139.6 (135.7–143.5)	1.30 (1.25–1.35)
**Gender**					
Men					
Matched Controls	14,967	963	110,806	86.9 (81.6–92.5)	1.00
Migraine cohort	14,967	1,274	108,781	117.1 (110.8–123.6)	1.35 (1.25–1.46)
Women					
Matched Controls	29,228	2,794	238,190	117.3 (113.0–121.6)	1.00
Migraine cohort	29,228	3,526	235,090	150.0 (145.1–154.9)	1.28 (1.23–1.34)
**Age of migraine diagnosis**					
40–59					
Matched Controls	18,948	303	183,685	16.5 (14.7–18.4)	1.00
Migraine cohort	18,948	409	183,332	22.3 (20.3–24.6)	1.35 (1.17–1.57)
60–					
Matched Controls	25,247	3,454	165,311	208.9 (202.2–215.7)	1.00
Migraine cohort	25,247	4,391	160,540	273.5 (265.7–281.3)	1.32 (1.26–1.38)
**Migraine aura**					
Matched Controls	44,195	3,757	348,996	107.7 (104.2–111.1)	1.00
Migraine without aura	16,628	1,898	136,279	139.3 (133.1–145.6)	1.29 (1.22–1.36)
Migraine with aura	4,004	456	33,883	134.6 (122.7–147.5)	1.24 (1.12–1.36)
Unspecified	23,563	2,446	173,709	140.8 (135.3–146.4)	1.32 (1.26–1.38)
**Time from migraine diagnosis**					
0–5 years					
Matched Controls	44,195	1,577	187,423	84.1 (80.0–88.3)	1.00
Migraine cohort	44,195	2,224	185,691	119.8 (114.8–124.8)	1.42 (1.34–1.51)
6–10 years					
Matched Controls	30,552	1,454	116,502	124.8 (118.5–131.3)	1.00
Migraine cohort	30,057	1,754	114,167	153.6 (146.5–160.9)	1.26 (1.14–1.4)
10 years or over					
Matched Controls	15,854	726	45,070	161.1 (149.6–173.1)	1.00
Migraine cohort	15,532	822	44,013	186.8 (174.3–199.8)	1.16 (1.05–1.28)

Patients with migraine had 1.29 times (HR, 1.29; 95% CI, 1.23–1.35), 1.35 times (HR, 1.35; 95% CI, 1.19–1.54), 1.36 times (HR, 1.36; 95% CI, 1.00–1.83), and 1.30 times (HR, 1.30; 95% CI, 1.17–1.45) higher risk of developing AD, VaD, mixed or other specified dementias, and unspecified dementia than their matched controls, respectively (Table 3).

**Table 3:** Risk of each type of dementia in the migraine cohort and matched controls

Variables	Subjects, No	cases, No	Person- Years, No	Incidence rate (95% CI) per 100,000 person years	Hazard ratio (95% CI)
**Alzheimer’s dementia**					
Matched Controls	44,195	2,685	348,995	76.9 (74.1–79.8)	1.00
Migraine cohort	44,195	3,401	343,871	98.9 (95.6–102.2)	1.29 (1.23–1.35)
**Vascular dementia**					
Matched Controls	44,195	408	348,995	11.7 (10.6–12.9)	1.00
Migraine cohort	44,195	544	343,871	15.8 (14.5–17.2)	1.35 (1.19–1.54)
**Mixed, or other specified dementias**					
Matched Controls	44,195	75	348,995	2.1 (1.7–2.7)	1.00
Migraine cohort	44,195	100	343,871	2.9 (2.4–3.5)	1.36 (1.00–1.83)
**Unspecified dementia**					
Matched Controls	44,195	589	348,995	16.9 (15.6–18.)	1.00
Migraine cohort	44,195	755	343,871	22.0 (20.4–23.6)	1.30 (1.17–1.45)

Sensitivity analyses showed that the association between migraine and all-cause dementia was significant when only migraine cases before the age of 60 were included. Patients with migraine also showed higher incidence of each type of dementia in sensitivity analyses. However, statistical significance was observed only for AD in analyses with 2-year washout period whereas VaD and unspecified dementia showed significant relations to migraine in analyses wtih 5-year washout period (Supplementary Table S1, S2).

## Discussion

In this study, we found that patients with migraine exhibited an increased risk of developing all-cause dementia, AD, VaD, and mixed or other specified dementias, and unspecified dementia compared to non-migraine-matched controls. Although the mechanism of this association remains largely unknown, there are several possible explanations for the pathways linking migraine and dementia.

First, migraine is associated with an increased risk of myocardial infarction and ischemic stroke, which are known risk factors for AD or VaD [15, 25]. Second, studies have found that migraine is related to structural and functional brain changes, including alterations in cerebral blood flow, increased white matter hyperintensities, subclinical infarct-like lesions, and brain volume changes [26–28].

Other possible mechanisms linking migraine and dementia include alterations in the cortisol-hippocampal pathway, inflammation, increased amyloid plaque formation, deficits in nerve growth factors due to comorbid depression, and chronic pain-related changes in the memory network structure of brain [29–31].

In the subgroup analyses, patients with migraine showed a higher risk of developing dementia than their matched controls, regardless of sex, age at migraine diagnosis, and follow-up duration. However, the magnitude of the association was prominent in patients diagnosed with migraine less than five years ago and attenuated with longer follow-up periods, indicating that a reverse causation (e.g., early diagnosis of dementia after consulting neurologists for migraine treatment) or a shared underlying cause between migraine and dementia may exist. Nevertheless, our findings provide evidence of longitudinal relationship between migraine and dementia development, since there was an increased risk of dementia in patients who had been diagnosed with migraine > 10 years ago or those who were diagnosed with migraine before the age of 60 years (when dementia was less prevalent).

Although the magnitudes of the association were found to be similar between MA and MO, more than half of the patients with migraine did not have information on aura. Moreover, since MA is known to have a higher risk of cardiovascular and cerebrovascular events, or structural abnormalities in the brain than MO, it is difficult to deny the theoretical relevance between MA and dementia with our findings alone [7]. Therefore, the impact of aura on dementia should be re-investigated with an accurate evaluation of migraine aura. Furthermore, future studies need to explore whether the characteristics of migraine attacks, such as severity or frequency, affect the development of dementia.

Contrary to our findings, most previous cohort studies did not find a statistically significant association between migraine and the risk of VaD [5, 32, 33]. The non-significant association may be attributed to the small sample size and lack of statistical power, because those studies showed consistent trends toward an increased risk of VaD in patients with migraine. Using a large sample with a nationwide cohort that provided sufficient statistical power, our study found that migraine was associated with an increased risk of VaD and AD. Moreover, we emulated the effect of prospective studies and reduced the immortal time bias in retrospective claims-based data using the risk-set matching method.

However, certain limitations of this study should be considered when interpreting its findings. First, several variables that could affect the development of dementia, such as educational level or baseline cognitive function, were not available for NHIS-HEALS. However, previous studies reported that educational level was not associated with migraine, or that individuals with higher educational levels were more prevalent with migraine [34, 35]. Additionally, we selected control individuals with the same baseline household income level as patients with migraine, and educational level was strongly correlated with income in South Korea [36]. Thus, in our study, educational level as a confounder had a minimal effect on the relationship between migraine and dementia. Second, the overall age of migraine onset among subjects in this study was greater than the peak prevalent age (35–39 years) of migraine because NHIS-HEALS comprised individuals aged 40–79 years in 2002. Therefore, a portion of the migraine cohort might comprise patients with active period of migraine instead of incident migraine cases. Although sensitivity analyses showed similar results to those of main analysis, findings for each type of dementia were not robust due to a small number of events. Third, because a large proportion of participants did not have information on presence of aura, we cannot draw a robust conclusion about the role of migraine aura on development of PD despite the evidence presented in previous studies and the biologically plausible mechanism. Last, the diagnosis of migraine, dementia, and other comorbidities may be inaccurate, owing to the inherent limitations of claim data. Therefore, to improve diagnostic validity and reduce false-positive cases, we required at least two diagnoses for migraine and at least one hospital admission or two outpatient visits for dementia and other comorbidities. Nevertheless, further studies involving longer follow-up durations (> 30 years), the entire adult population and detailed information regarding migraine are required to enhance the generalizability of our findings.

## Conclusion

In conclusion, in the present study, patients with migraine had an increased risk of developing all-cause dementia, AD, VaD, and other dementias compared with their risk-set matched controls. However, further studies are warranted to generalize our findings and elucidate the underlying pathophysiological mechanisms linking migraine and dementia.

## Supplementary Information

**Additional file1**: **Table S1. **Association between migraine and risk of dementia among individuals who diagnosed migraine before age of 60. **Table S2. **Association between migraine and risk of dementia among with five years of washout period among individuals who diagnosed migraine before age of 60.

## Data Availability

The datasets generated and/or analyzed during the current study are not publicly available because the NHIS exclusively allows authorized persons to access data in a separate space.

## Abbreviations

AD: Alzheimer’s dementia
VaD: Vascular dementia
MA: Migraine with aura
MO: Migraine without aura
NHIS-HEALS: Korea National Health Insurance Service Health Screening Cohort
NHIS: National Health Insurance Service
ICD-10: International Statistical Classification of Diseases and Related Health Problems, 10th revision
BMI: Body mass index
IR: Incidence rate
CI: Confidence interval
HR: Hazards ratio
