# Supplementary material for: Increased risk of all-cause, Alzheimer’s, and vascular dementia in adults with migraine in Korea: a population-based cohort study
Source: J Headache Pain. 2022 Aug 24;23(1):108. doi: 10.1186/s10194-022-01484-y (PMC9404580; doi:10.1186/s10194-022-01484-y)
Supplement: Supplementary file 1 — Additional file1: Table S1. Association between migraine and risk of dementia among individuals who diagnosed migraine before age of 60. Table S2. Association between migraine and risk of dementia among with five years of washout period among individuals who diagnosed migraine before age of 60. [file 10194_2022_1484_MOESM1_ESM.docx]

**Supplementary Materials**

**Table S1.** Association between migraine and risk of dementia among individuals who diagnosed migraine before age of 60

**Table S2.** Association between migraine and risk of dementia with five years of washout period among individuals who diagnosed migraine before age of 60

| **Table S1. Association between migraine and risk of dementia among individuals who diagnosed migraine before age of 60** | | | | | | |  |  |
| --- | --- | --- | --- | --- | --- | --- | --- | --- |
| **Variables** | | **Subjects,**  **No.** | **cases,**  **No.** | **Person-**  **Years, No.** | **Incidence rate (95% CI) per 100,000 person years** | **Hazard ratio (95% CI)** |  |  |
| **All-cause dementia** | | |  |  |  |  |  |  |
|  | Matched Controls | 18,948 | 303 | 183,685 | 16.5 (14.7–18.4) | 1.00 |  |  |
|  | Migraine cohort | 18,948 | 409 | 183,332 | 22.3 (20.3–24.6) | 1.35 (1.17–1.57) |  |  |
| **Alzheimer’s dementia** | | | | | | |  |  |
|  | Matched Controls | 18,948 | 163 | 183,685 | 8.9 (7.6–10.3) | 1.00 |  |  |
|  | Migraine cohort | 18,948 | 242 | 183,332 | 13.2 (11.6–15.0) | 1.49 (1.22–1.81) |  |  |
| **Vascular dementia** | |  |  |  |  |  |  | |
|  | Matched Controls | 18,948 | 60 | 183,685 | 3.3 (2.5–4.2) | 1.00 |  |  |
|  | Migraine cohort | 18,948 | 74 | 183,332 | 4.0 (3.2–5.1) | 1.24 (0.88–1.74) |  |  |
| **Mixed, or other specified dementias** | | | |  |  |  |  | |
|  | Matched Controls | 18,948 | 8 | 183,685 | 0.4 (0.2–0.9) | 1.00 |  |  |
|  | Migraine cohort | 18,948 | 11 | 183,332 | 0.6 (0.3–1.1) | 1.38 (0.55–3.42) |  |  |
| **Unspecified dementia** | | |  |  |  |  |  |  |
|  | Matched Controls | 18,948 | 72 | 183,685 | 3.9 (3.1–4.9) | 1.00 |  |  |
|  | Migraine cohort | 18,948 | 82 | 183,332 | 4.5 (3.6–5.6) | 1.14 (0.83–1.57) |  |  |

| **Table S2. Association between migraine and risk of dementia with five years of washout period among individuals who diagnosed migraine before age of 60** | | | | | | |  |  |
| --- | --- | --- | --- | --- | --- | --- | --- | --- |
| **Variables** | | **Subjects,**  **No.** | **cases,**  **No.** | **Person-**  **Years, No.** | **Incidence rate (95% CI) per 100,000 person years** | **Hazard ratio (95% CI)** |  |  |
| **All-cause dementia** | | |  |  |  |  |  |  |
|  | Matched Controls | 10,697 | 84 | 72,259 | 11.6 (9.4–14.4) | 1.00 |  |  |
|  | Migraine cohort | 10,697 | 128 | 72,119 | 17.7 (14.9–21.1) | 1.53 (1.16–2.01) |  |  |
| **Alzheimer’s dementia** | | | | | | |  |  |
|  | Matched Controls | 10,697 | 58 | 72,259 | 8.0 (6.2–10.4) | 1.00 |  |  |
|  | Migraine cohort | 10,697 | 71 | 72,119 | 9.8 (7.8–12.4) | 1.23 (0.87–1.74) |  |  |
| **Vascular dementia** | |  |  |  |  |  |  | |
|  | Matched Controls | 10,697 | 13 | 72,259 | 1.8 (1.0–3.1) | 1.00 |  |  |
|  | Migraine cohort | 10,697 | 29 | 72,119 | 4.0 (2.8–5.8) | 2.24 (1.16–4.30) |  |  |
| **Mixed, or other specified dementias** | | | |  |  |  |  | |
|  | Matched Controls | 10,697 | 1 | 72,259 | 0.2 (0.1–0.3) | 1.00 |  |  |
|  | Migraine cohort | 10,697 | 3 | 72,119 | 0.3 (0.2–0.4) | 3.01 (0.31–28.93) |  |  |
| **Unspecified dementia** | | |  |  |  |  |  |  |
|  | Matched Controls | 10,697 | 12 | 72,259 | 1.7 (0.9–2.9) | 1.00 |  |  |
|  | Migraine cohort | 10,697 | 25 | 72,119 | 3.5 (2.3–5.1) | 2.09 (1.05–4.16) |  |  |
|  | | | | | | |  |  |
